# Analyzing Patterns in NewSTEPs Site Review Recommendations: Practical Applications for Newborn Screening Programs

**DOI:** 10.3390/ijns5010013

**Published:** 2019-02-12

**Authors:** Yvonne Kellar-Guenther, Marci K. Sontag, Eric Linder, Sikha Singh, Ruthanne Sheller, Jelili Ojodu

**Affiliations:** 1 Center for Public Health Innovation, CI International, 7852 S. Elati St., #201, Littleton, CO 80120, USA; 2 Colorado School of Public Health, University of Colorado, Anschutz Medical Campus, Aurora, CO 80045, USA; 3 Healthgrades. 1801 California Street #800, Denver, CO 80202, USA; 4 Association of Public Health Laboratories, 8515 Georgia Avenue, Suite 700, Silver Spring, MD 20910, USA

**Keywords:** newborn screening, continuous quality improvement, evaluation, technical assistance, public health, site reviews

## Abstract

The Newborn Screening Technical assistance and Evaluation Program (NewSTEPs) conducts non-regulatory site reviews of state newborn screening programs in the US with the goal of providing comprehensive reports and recommendations to support quality improvements within the system. A detailed coding and qualitative analysis of data extracted from reports of seven programs visited between 2012 and 2017, of thirteen pre-site visit surveys completed by state newborn screening programs, and of information from interviews conducted with three site review experts revealed four common themes that exist across states within the national newborn screening system. These themes include opportunities to implement improvements in: (1) communications inside and outside of the state newborn screening program, (2) education, (3) information technology, and (4) operations. The cross-cutting recommendations provided by NewSTEPs within the comprehensive site review reports may prove valuable for all state programs to consider and to incorporate as quality improvement measures in the absence of a full site review. The analysis of the site review process and recommendations identified important opportunities for improvement, many of which were previously unknown to be common across programs, and also provided affirmation of known challenges.

## 1. Introduction

Newborn screening (NBS) is a public health program designed to decrease or eliminate newborn morbidity and mortality from some genetic disorders through pre-symptomatic identification [1,2]. In the United States (US), NBS is a complex system of laboratory testing of dried blood specimens and point of care screening for hearing and congenital heart disease, utilizing a network of professionals in birthing facilities, state public health laboratories, state health programs, and specialty care centers [3]. To manage the complexities of the newborn screening systems and to be effective and efficient, US state newborn screening programs may require technical assistance [4,5].

The Newborn Screening Technical assistance and Evaluation Program (NewSTEPs) provides a comprehensive system of resources and technical assistance for NBS programs in the US. NewSTEPs is a program of the Association of Public Health Laboratories (APHL) and is funded by the US Department of Health and Human Services (HHS), Health Resources and Services Administration (HRSA), and the Maternal and Child Health Bureau (MCHB) [6]. NewSTEPs conducts non-regulatory site reviews in response to state NBS program requests [6,7]. Every site review covers the following areas: Organizational Structure, Newborn Screening Funding, Legislation and Policy, Ethics, Emergency Preparedness, Laboratory Systems, Testing Results, Lab Quality Assurance/Quality Control (QA/QC), Specimen Transport, Short-Term Follow-Up, Point of Care Screening, Long-Term Follow-Up, Short-Term Follow-Up QA/QC, Education, Birthing Facilities, and Information Systems. If the state has an additional need, the site review is modified to ensure that need is also addressed. This report summarizes key findings from site reviews, with the goal to provide a broad application of quality improvement efforts nationwide, even in the absence of state-specific site reviews.

## 2. Materials and Methods 

### 2.1. Site Reviews

US state or territorial NBS programs requested a site review from NewSTEPs through a formal process, including a letter of request from an authorized official within the state. NewSTEPs assembled site review teams (SRT) comprised of peer experts from across the nation, spanning areas of expertise within the NBS system including laboratory, follow-up, and clinical specialist experts. Additional site review team members with expertise in information systems were added when needed. To prepare for the site review the SRT: (a) reviewed the information provided by the states (online Supplement 1, Question 9) including the NewSTEPs pre-site review form, (b) participated in a pre-site visit training that highlighted the typical site review process and provided an overview of their role in that process as well as expectations, and (c) met with the key staff at the requesting state program at least once by conference call prior to the on-site review. 

Recognizing that NBS is a complex system requiring many critical partners, once on-site, the SRT meets with public health leadership, state NBS leadership, NBS staff, birthing center staff from at least two birthing facilities, and any clinical specialists who work with the program who are invited by the state program to take part in the site review. [8] The visit extends 3–5 days depending upon the population of the state, the geographic location of the laboratory and follow-up staff, and the needs highlighted in the pre-site review form. Each site review starts with an overview presentation by the NBS program. The NBS program is asked to cover the 16 areas that will be covered in-depth during the site review along with any additional concerns they have. In addition to asking customized questions based on pre-identified needs, the team asks questions organized in a site review manual encompassing state legislation and policy, ethics, funding models, organizational structure, emergency preparedness, laboratory systems, follow-up systems, blood spot and point of care testing, education, and information systems (online Supplement 2). The site review team also observes the state NBS program’s workflow, processes, relationships between staff as well as with other NBS stakeholders as well as the workflow and processes of two birthing centers. Site review team members are asked to be familiar with each section of the manual. Every night the team debriefs to discuss observations and whether all the information is complete or whether additional questions need to be answered for each of the 16 areas covered. While the site review manual has been updated based on feedback from the SRT, all site reviews conducted by NewSTEPs have included the same 16 areas.

At the conclusion of the on-site portion, the SRT meets with key personnel to highlight key observations and provide an overview of the next steps. Following the visit, the NewSTEPs SRT provides a comprehensive written report that summarizes findings from the visit including general perceptions, an assessment of the functions of each component of the screening program (pre-analytical, analytical, and post-analytical), needs identified by the SRT, and a list of recommendations for future program changes and growth. NBS programs are encouraged to share the report with programmatic and state leadership to identify opportunities for future resource allocation and quality improvement activities. Approximately 6 months following the site review, NewSTEPs requested that the NBS program complete a follow-up survey (online Supplement 3) to identify changes that occurred as a result of the site review and to get feedback from the program on the site review process. This step was added with the third site review. To date three states have completed this survey. The first two programs that received a site review presented a joint poster at a national NBS meeting that addressed the tangible impacts [9]. 

### 2.2. Data Analysis

We reviewed seven full site review reports, thirteen pre-site review documents completed by laboratory and follow-up programs in the seven NBS programs who had site reviews, interviews with three SRT members who had been to multiple states, three completed post-site review surveys, and one poster presented on the results of the site review on two states [9]. Two authors coded the site review reports using a content analysis approach to identify patterns across NBS programs. Larger categories in the codebook mimicked the sections of the site review report—Organizational Structure, Legislation and Policy, Ethics, Newborn Screening Funding Needs, Lab System Needs, Specimen Transport, Testing Results, Emergency Preparedness, Short-Term Follow-Up, Short-Term Follow-Up Quality Assurance/Quality Improvement, Birth Facility, Point of Care Screening, Education, and Information Systems. Themes within each category were identified inductively, utilizing a constant-comparison approach. 

The primary coder reviewed and coded all seven site review reports, of which five were final reports, and two were draft forms. A second coder reviewed four of the seven site review reports early in the process to standardize coding. The two reviewers discussed any coding discrepancies and addressed them through conversation. After analyzing the seven site review reports, the second coder coded all thirteen pre-site review forms to identify any needs not captured in the final reports. No new codes were added. The primary coder then interviewed and coded the responses of three experts—one laboratorian, one follow-up specialist and one clinician—who had been on at least two site reviews. No new codes were added. All reports were re-visited after the coding scheme was finalized. Themes that were mentioned in at least four of the seven site reviews are included in the findings. Finally, the degree of overlap between pre-site visit reports and the site review was calculated by comparing the areas where the SRT noted the most needs to the areas of need noted by the state program staff. 

## 3. Results

NewSTEPs conducted seven site-reviews visited between 2012 and 2017: two were large states (over 200,000 births per year), two were medium states (between 80,000 and 200,000 births per year), and three were small states (less than 60,000 birth per year). The SRT was on site for 3–5 days per state. 

The sections of the site review manual were used to organize needs and themes for each program. These sections became the categories, and within each category are the types of needs identified by the SRT. Four main themes emerged that cut across the categories of review: communication inside and outside the state NBS program (identified at all sites and by all experts); education (identified at all sites and by all experts); information technology (identified at 6 sites and by 2 experts); and operations (identified at all sites and by all experts). Findings from the site reviews were qualitatively analyzed, and themes are presented. Programmatic changes reported by state NBS programs that resulted from the site review are listed at the end of each theme. 

### 3.1. Communication within the State Newborn Screening Program

Site review reports for all seven programs highlighted the need to improve communication between the laboratory and follow-up staff (Table 1). Four states had also noted that communication was problematic in their pre-site review forms. One frequently identified need was better communication within the program across organizational structure, lab systems, and short-term follow-up sections of the site review reports. “Seems to be there is some lack of communication from Lab to Long-term follow-up (LTFU); specifically, they stopped testing for [condition] which had an impact on the LTFU team, but [the lab] did not notify them of this change” (site review report). Site reviewers also noted that for a few programs, the lack of communication between parts of the state NBS program posed challenges for quality assurance processes because the lab was not getting feedback from follow-up that could affect cut-off values.

The lack of communication between Laboratory Information Management Systems (LIMS) and short-term follow-up IT systems noted in both the pre-site visit and the site review report causes delays in result reporting. Follow-up staff wait for a report from the laboratory on out-of-range results, and in some cases, need additional information to call out the results to the provider. For example, one reviewer wrote, “Although it takes less than three days to complete all testing, there are four to five days of delays to report out results after all testing are completed. These delays, are caused by limitations on staff and lack of a centralized LIMS system.” The lack of communication between the two information systems also makes it difficult for the laboratory to record the final diagnosis in the laboratory system. 

In post site-review surveys, states reported the following activities were implemented to improve communication:

- The laboratory and follow-up teams began meeting more regularly. One state reported the two teams meet two times a month while another state reported that the teams meet weekly but check in daily for tactical issues.
- The laboratory supervisor working on Saturday began notifying the follow-up specialist if critical results are anticipated.
- To address the LIMS issues, one state team reported they are considering options for new LIMS/follow-up program application with the goal of increasing transparency between the two areas and increasing timeliness.

### 3.2. Communication Outside the State Newborn Screening Program

While states identified internal communication needs, they did not identify communication problems with clinicians or birthing centers on their pre-site review forms. The SRT, however, noted communication problems between the state NBS program and birthing centers and specialists as a top need in the site review reports. The SRT met with at least two birthing centers and any specialists invited by the state NBS program to be part of the site review. Birthing facility staff in four states expressed an interest in receiving information about the number of newborns from their facility identified with a condition through NBS (Table 2). In one state, hospital staff said they were not told which specimens were “poor quality” or why. The hospital staff in this state reported that they were frustrated they learned of issues on a timeliness report and then had to find a report on the state program’s website rather than being notified right away with more detail. Finally, while NBS programs reported they were providing information to birthing facilities on blood spot specimen collection and shipping issues, the staff at the birthing facility were not clear who was receiving this follow-up information; staff who collected and/or were in charge of shipping the specimens were not always the ones who received this information. “Specimens are collected by the nurses in the NICU, but they do not receive copies of the report card [notes how well the program is doing in terms of timeliness of collection, the number of unsatisfactory specimens] which is only sent to the hospital lab” (site review report).

Clinical specialists in five states who met with the SRT expressed their desire to communicate more with the NBS program and be part of the quality assurance process (Table 2). Specialists in some of the programs also requested that the state NBS program asks for their input on the ACT/FACT [10] sheets used in their states and that these sheets be updated when new information is available about the condition or treatment of the condition. 

### 3.3. Birth Provider and Facility Education

While a need for better education for birthing providers and facilities only came up in one pre-site review form, it was a strong theme in all seven site review reports (Table 3). The most frequently mentioned educational need was to ensure birthing facilities and providers had and were using current NBS educational materials. Sometimes these audiences knew their materials were out-of-date but did not know how to get current materials; whereas, in other cases, the audience did not realize the information was no longer current. Reviewers noted that outdated resources were often being used at birthing facilities because groups had to actively look at the NBS program website and seek out new materials, and the facility staff had not realized newer materials were available. One reviewer stated it “was noted that the [state] NBS program websites are difficult to navigate and that [hospital] staff would like easier access to education materials.” 

In addition to the need for up-to-date print materials and training videos, hospital staff in six sites asked for more NBS training. “Staff is very interested in received educational interventions to learn more about NBS and in quality improvement projects around specimen quality, timeliness, and outcomes” (site review report). NBS state policy revisions combined with new birthing providers and facility personnel results in hospital staff without current information. Birthing center staff requested not just more training, but also more consistent training for their staff (e.g., knowing that the staff will get trained every year). For example, many birthing center staff could not recall the last formal training they had received by the state NBS program while others indicated it had been a few years. “They [birthing facility] have not had an in-service or outreach from the state in the past 2–3 years and think it would be helpful to have a visit.” Training topics that came up in more than one state are as follows: (a) when to talk to parents about NBS, (b) how to document parent refusal, (c) that parents could, in fact, refuse NBS, (d) best practices for blood spot specimen collection and drying, (e) when the courier picks up from their facility, and (f) how to follow the critical congenital heart disease (CCHD) screening protocol. Reviewers in two states also noted there was a need to educate birthing providers who provide point-of-care screening why it was important that they report the screen results to the NBS program.

In post site-review surveys, states reported the following activities were implemented to improve communication between the program with specialists and birthing centers:

- Formed a newborn screen advisory committee that had not previously been in place.Published an ongoing newsletter to communicate more regularly with birthing centers.
- Created and started sending birthing center report cards so birthing centers can track their timeliness for specimen collection, their percentage of unsatisfactory specimens, etc.
- Began training across the state/region on proper sample collection.
- Worked with specimen submitters to improve the transit time of their specimens to the state laboratory.
- Explored hiring an educator who can create and provide hospitals with brochures, collect and share patient stories as part of training staff who will collect the initial NBS, and/or sending feedback to a birthing center when an infant born in their center was identified as having positive newborn screen.
- Worked with a metabolic specialist to create a rationale document for early collection of NBS specimens for the NICU population.
- Began a program where birthing facility staff and other submitters tour the state NBS laboratory and the follow-up program so these submitters can see the “big picture”.

### 3.4. Information Systems and Information Technology Needs

Information systems and IT needs were identified in the ethics, laboratory system, short-term follow-up, and IT section of the site review reports. In addition to laboratory and follow-up IT systems that could communicate, reviewers identified additional IT needs in six of the seven states (Table 4). Two sites specifically asked for IT consultation as part of the site review. In these sites, the team discovered that the state NBS program was not aware of all the features they could utilize with their current LIMS. Reviewers in four states and two interviewees noted that the information management systems utilized by some laboratories were not designed for lab assessments and therefore made it difficult for the labs to use. In some states, the review team suggested the state program upgrade their LIMS.

The site reviewers identified the need for a better system to record if all newborns received complete screens in 5 of 7 states, including tracking parental refusals, locating missing specimens and documenting infants lost to follow-up. Site-reviewers identified this need, while the pre-site review reports did not. Site reviewers noted, “There is no formal tracking for missing specimens since there is not a way to determine if one is missing,” and “Refusals are documented on the card, but without a known denominator, it is hard to determine how many are refusing.”

Three out of seven site review reports identified the need to create a linkage between NBS systems and the state’s electronic birth records to calculate a reliable denominator for quality assurance analysis. Access to the systems for all weekend/holiday/after house program staff via a virtual private network (VPN) in case of an emergency was another need noted in three of the seven states. Additionally, reviewers noted that short-term follow-up programs need to document in an electronic information system rather than relying on a paper system. Reviewers in two state site reviews noted that the state’s reliance on paper documentation slowed down the process and could make response during an emergency difficult if the team had to move off-site. 

In post site-review surveys, states reported the following activities were implemented to address IT needs identified in the site review report:

- Hired two new LIMS staff to support current LIMS administrator.
- Upgraded LIMS from outdated to current.
- Evaluated new LIMS systems to upgrade or select new vendor to meet program’s needs.
- Developed a provider portal which provides access to medical professionals who could access laboratory reports for their patients.
- Joined the APHL HIT user group based on the LIMS they are using.
- Implemented a module that will allow the program to match NBS records with vital records.

### 3.5. Operations in State Newborn Screening Programs

The need to strengthen how the state NBS programs are conducting screening and providing follow-up care, as well as the state NBS program resource needs was the final theme identified.

#### 3.5.1. Newborn Screening State Program Standard Operations Procedures, Regulations, and Rules

Programs completing the pre-site review forms were less likely to identify procedural and regulatory needs than the site reviewers. Specifically, all site review reports identified a need to update policies and rules, while none of the pre-site review forms identified this. A financial assessment of the program, reassessing fees and billings was noted in five site review reports but in only one pre-site review form. Similarly, pre-site review forms did not highlight blood spot retention and storage policies as a need, but five site review reports noted this need. Site review teams noted in a few states there was no continuity of operations plan (COOP) and in several other sites the plan existed, but the NBS program had not recently practiced the plan. 

The state NBS team noted the low birth weight/prematurity algorithm needed review, including identifying procedures for newborns receiving total parenteral nutrition (TPN). Reviewers noted that programs might miss affected newborns due to collections occurring too early or otherwise in conflict with existing best practices. 

All seven site review reports noted that the state program needs to annually review their standard operating procedures (SOPs), regulations, and rules (Table 5). “This yearly review allows programs to ensure program staff are familiar with SOPs, regulations, and rules but also enables programs to think about needed changes.” For example, reviewers suggested programs have SOPs that include guidance on (a) establishing the age of the specimen, (b) special precautions for shipments where the mother has HIV, (c) tracking specimen receipt/monitoring the specimen, (c) blood spot retention, (d) follow-up notification, and (e) closing a case where there is no repeat screen for an abnormal result.

In post site-review surveys, states reported the following activities were implemented to improve program standard operating procedures, rules and regulations:

- Revised newborn screening regulations.
- Changed their state policy so that “protein feed for at least 24 hours” is no longer required.
- Updated their emergency preparedness continuity of operations plan (COOP)
- Reviewed forms, policies, and processes being used by other states to influence a specimen destruction plan.
- Had data destruction conversation with LIMS vendor.
- Developed forms that parents can use to request specimens be destroyed after testing.
- Explored implementing a newborn screening fee and a merged panel.
- Worked with state legislature to re-introduce legislation to get the fee changed.
- Requested and were granted a fee increase.
- Sought a procurement exemption from the Governor. This led to the state program being able to secure a contract for equipment, reagents, and services for NBS testing in two months instead of two years.

#### 3.5.2. Dried blood Spot Screening Algorithms, Cut-Offs, and processes 

Reviewers identified the need for programs to strengthen their sample testing algorithms, cutoffs, and testing processes in six out of seven states (Table 5). Reviewers noted that utilizing second-tier testing could improve the specificity of tests. Re-evaluating cutoffs, especially related to the age of the infant and special circumstances, may also improve test accuracy. One site review report noted that metabolic specialists were frustrated with a recent cutoff change that resulted in too many false positives for a specific disorder, further supporting the need to continuously evaluate cutoffs within a program. Further, the site review report identified opportunities for using newer testing technology and approaches, which may result in more sensitive and specific results. Both site review reports and the pre-site review tool identified the need for space, equipment, and ongoing maintenance of laboratory resources. 

The other need identified within the laboratory is to have a process in place to ensure efficient collection of repeat specimens on infants with an unsatisfactory specimen or out-of-range result. In one report, reviewers noted that it could take the state program up to 14 days from the request for a repeat specimen to receipt of that specimen by the lab. Further, reviewers noted that some programs could report critical results more expeditiously with timelier specimen processing and result reviewing. 

Finally, a challenge identified universally is the hiring, training, and retention of qualified laboratory personnel. Reviewers noted that, “Both the lab and follow-up services are understaffed and have had a hard time retaining strong employees because of the inability of the units to reallocate staff to higher level positions and provide well-deserved raises.” They also noted, “the process to add new positions/human resources within the NBS laboratory is lengthy and requires approval from the Commissioner.” 

In post site-review surveys, states reported the following activities were implemented to address dried blood spot screening algorithms, cut-offs, and processes:

- Had senior level staff provide confirmation on qualifying criteria for specimen rejections.
- Worked with the LIMS provider to configure “test specific unsatisfactory specimen” which will be applied only at the disorder level and not specimen level.
- Evaluated condition specific cut-offs and conducted new statistical analysis to monitor cut-off changes for several conditions including a plan for surveillance of cut-off variation for state’s presumed positives population on a yearly basis.
- Hired a Quality Assurance Officer who is located in laboratory administration to ensure transparency in the quality management system.
- Added a refusal section to the NBS module of the birth certificate registry so hospitals can report refusals to the NBS program.

#### 3.5.3. Follow-up Processes

Needs identified in the follow-up program parallel those identified in the laboratory in identifying newborns who were not screened and those who need a repeat screen. Some reviewers noted that programs do not complete active follow-up for newborns with equivocal or unsatisfactory results and that some programs need to develop formal tracking for missing specimens. Further, reviewers noted that there should be a better distinction between the repeat specimen process and the cases lost to follow-up. Finally, site reviewers noted that some follow-up programs could benefit from identifying procedures that are disorder specific following an abnormal result notification. 

The site reviewers noted in four site review reports that the program needed more follow-up staff, while only one state’s pre-site review forms identified this need. Specific needs included staff who were able to efficiently utilize the LIMS systems to extract information needed for follow-up staff. Further, site reviewers suggested that programs needed specific Quality Assurance personnel to help with identifying areas for improvement within the systems. Similar to laboratory staffing, reviewers noted that retention of follow-up staff was difficult because there was a lack of opportunities for advancement in the NBS programs. 

In post site-review surveys, one state reported that the site review recommendations played an important role in helping them acquire needed resources. “Lack of follow-up on [for] babies more than 30 days old has provided justification to hire one additional FTE in follow-up for current workload.” (post-evaluation survey response). Other activities states reported implementing to improve the follow-up process included:

- Hired new staff.
- Worked on a follow-up algorithm for unsatisfactory specimens.
- Created NBS follow-up process maps to outline procedures for initial notification of all abnormal results.
- Follow-up staff worked with human resources to explore the option of higher title/rank for follow-up staff to increase pay and provide opportunities for promotion.
- Hired a Quality Improvement specialist.
- Initiated a quality improvement project to promote voluntary enrollment in Early Intervention after the diagnosis of a hearing loss.
- Built a module so that hospitals enter CCHD data within the birth certificate registry.

### 3.6. Barriers to Addressing Needs Identified by Site Review Team

All five of the states who provided input after the site review were able to share many of the activities they have started, and in some cases completed, as a result of the site reviews. Of these five states, two provided some insight into the barriers they face in addressing many of the needs identified by the SRT. Time was identified as a barrier. States needed time to hire and on-board staff to tackle recommendations made by the SRT, and time to procure a contract to resources such as new LIMS. One state also reported they had to delay addressing SRT recommendations because they needed to make changes to their LIMS for at least one new disorder.

Another barrier identified by the two states was the rules and/or legislation process. One state mentioned that in order to meet a recommendation, the state would need an administrative rule change to provide tracking and surveillance for point-of-care testing. This same state indicated that the state legislature did not allow the use of dried bloodspots for research so they were unable to meet a SRT recommendation. The time delays due to a slow legislation process was also mentioned as a barrier. One program said they were waiting for a bill to be introduced in state legislation.

A final barrier was that the recommendations were “resource intensive”. One state felt that availability of grant funds to purchase needed resources, access to tools that can be used to address the SRT’s resource heavy recommended strategies, and/or connections to other states of similar size/capacity who could share what they have done to meet these needs would help overcome this barrier. This last point is supported by another state who said “the connections provided during the site visit were useful and we have been making contact with other states as well who have been more than willing to share their information” (quote from state, post-site review survey). 

### 3.7. Intersection of Needs Articulated and Needs Identified 

**Figure 1:**
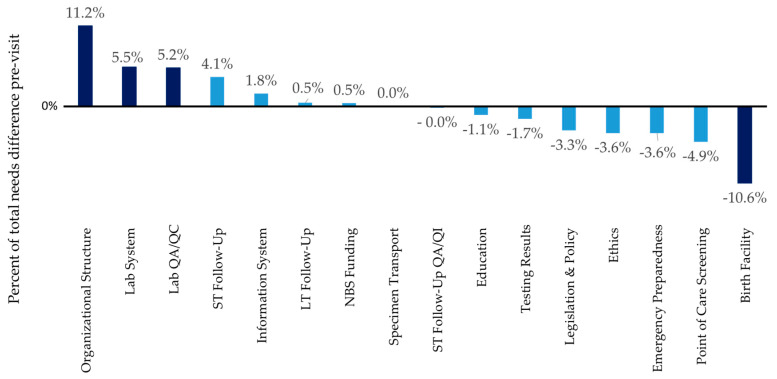
Comparison of frequency of needs identified by states in the pre-site review form vs the frequency of needs identified by the site review team.

The goal of this report was to provide an overview of needs and solutions identified in a subset of the NBS programs so that other programs may benefit. When site reviewers identified needs that were not identified by the state program, these insights may help other programs to find challenges that are less readily apparent in a self-assessment. While there is an overlap between the state NBS staff identified needs and those identified by the SRT, there were additional needs identified. “[The site review team] gave very good suggestions on areas where we can make improvements to our program, including areas we hadn’t considered or were even aware were things that other states are doing” (quote from state, post-site review survey). The three areas with the most needs identified by the SRT and the state NBS program prior to the site review include the laboratory system (64 instances in report; 16 in pre-site form), followed by short-term follow-up (48 instances in report; 12 in pre-site form), and organizational structure (46 instances in report; 17 in pre-site form). Birth facility needs ranked second for the most needs identified in a site review report (49 times), but this need was only mentioned once in the pre-site visit form suggesting that sites may be unaware of this need. Figure 1 illustrates the difference in needs mentioned in the final report vs in the pre-site visit form. Overall, needs identified by the state on the pre-site visit tool (represented as percent of total needs identified) were in good agreement with needs identified by the site review team in the report (<5% difference) for 12 of the 16 reported areas (Figure 1). The darker bars in Figure 1 represent areas where there is more than 5% disagreement between what the NBS programs felt they needed and what the site reviewers felt the programs needed. Organizational Structure, Lab System, and Lab QA/QC were more frequently cited as a need by the state in the pre-site visit report (11%, 5.5%, and 5.4% difference, respectively) (See Figure 1, darker bars represent the areas where disagreement is above 5%). Birth facility needs were more frequently identified by the site review team; the site review teams noted 49 needs around birthing facilities vs the one need mentioned by a state on the pre-site visit form (10% difference) (See Figure 1, darker bars represent the areas where disagreement is above 5%).

Agreement between the site review report and pre-site visit form may be by design. The SRT reads the pre-site review forms in preparation for the visit. Further, the pre-site visit form can shape who is part of the SRT. This flexible approach fits within the site review typology posited by Haynes et al. [11] in which site reviews can range from exploratory site reviews (no standard process) to highly standardized and detailed protocol. To remain sensitive to the needs of the state NBS program while attempting to minimize bias, NewSTEPs uses a semi-structured approach, with consistent structure surrounding the on-site reviews. To encourage a thorough review, the SRT always meets with NBS program staff and clinical specialists and visits at least two birth centers at each visit. Site reviewers are also given a site review manual created by the NewSTEPs Evaluation Workgroup, informed by the Clinical and Laboratory and Standards Institute (CLSI) Newborn Screening Guidelines [12,13,14,15,16,17,18] and the Performance and Evaluation Assessment Scheme, [7] and edited based on observational data during site reviews and interviews with SRT members following a site review. Use of the manual and nightly debriefs among the SRT ensure adequate discussion of all topics on each site review. The consistent structure of each on-site review may explain the discordance between the pre-site review and the final site review reports.

## 4. Discussion

The systematic review of the site reviews revealed several common themes. The site review teams proposed several recommendations to help address the identified needs:

(I) Systematic communication within the program: “there is a need for regular (e.g., monthly, bi-monthly, quarterly) meetings with laboratory and follow-up staff.” During regularly scheduled meetings, the program could discuss programmatic roles, decisions, processes, and workflows. The programs could also discuss abnormal results and false positive rates. Both laboratory and follow-up staff should take a comprehensive tour of each other’s units.
(II) Improving communication between the information systems used by laboratory and follow-up staff: Access between laboratory and follow-up teams’ LIMS systems can support data exchanges which will lead to quicker reporting of screening results and quality assurance efforts.
(III) Communication with clinical specialists: Invite clinical specialists to meet with the state program and provide input. The input may include how the program sets cut-offs and the impact NBS has on their clinic’s work.
(IV) Communication and education with stakeholders external to the state NBS program: (1) Notify appropriate people at the birthing facility of out-of-range results and unsatisfactory specimens; (2) Communicate with the entire birthing facility staff involved in any part of NBS, those involved in specimen collection through specimen transport; (3) Notify birthing centers and clinicians about new educational materials and simplify the process to access the new materials; and (4) Educate birthing providers who provide point-of-care screening about the importance of reporting results to the NBS program.
(V) Information technology: (1) Work with LIMS vendors or other programs using the same platforms to try to maximize the power of the LIMS; (2) Consider an upgrade to the LIMS, if warranted; (3) Track refusals electronically; (4) Connect NBS results with state electronic birth records; and (5) Grant virtual private network (VPN) access to all weekend/holiday/after-hours program staff in case of an emergency.
(VI) Standard operating procedures: State NBS programs should review their standard operation procedures (SOPs), regulations, and rules annually. Specifically: (1) Develop processes and procedures to identify newborns not screened, track parent refusals, track repeat screens; and (2) develop a process to ensure efficient collection of repeat specimens following an unsatisfactory initial specimen.
(VII) Continuity of operations plan (COOP): Programs without a COOP should talk to other states and NewSTEPs staff to help them identify the components of a COOP. Once the plan is in place, state programs need a procedure to test the plan annually. The whole state NBS system, including birthing facilities, should be part of the testing.

## 5. Conclusions

NBS program site reviews can provide affirmation of the known challenges and needs while uncovering factors that were previously unknown to the program. Although the purpose of site reviews is to provide feedback to the state NBS programs, site review team members also learned from the state NBS programs they reviewed. Through the site review process, reviewers identify needs in their own programs as well as identify practices to strengthen their programs. 

The assimilation of lessons learned from site reviews provides other NBS programs insight into systematic challenges within the US. NBS programs who have not had a site review may recognize the needs identified and may find that the recommendations could strengthen their own programs. States can also conduct internal reviews using the NewSTEPs site review manual (online Supplement 2) or request customized self-reviews through NewSTEPs.org. 

NewSTEPs continually assesses the site review process with each site review team to ensure the reviews are as beneficial and efficient as possible. The reviews will continue to evolve to provide the expertise and technical assistance identified by the site review report, either directly or through connections with other experts that can help them address their needs. 

## Figures and Tables

**Table 1:** Needs identified in State Newborn Screening Program Site Reviews: Communication within the Newborn Screening Program: themes identified (Number of times reported in site review report, individual interview, and pre-site review forms).

Category	Sub-Theme
Lab and follow-up need better communication (7/7 reports, 2/3 interviews, 4/7 pre-site review forms)	
	Need to meet regularly to share ideas/provide feedback
	Improve communication between both parts of the program around continuous operational activities
	Share changes to follow-up or lab with other part of newborn screening program
Lab and follow-up need to have the same chain of command/leadership (5/7 reports, 0/3 interviews, 1/7 pre-site review forms)	
Lab and follow-up IT systems need to be able to communicate (7/7 reports, 0/3 interviews, 7/7 pre-site review forms)	

**Table 2:** Needs identified in State Newborn Screening Program Site Reviews: Communication Outside the State Newborn Screening Program themes identified (Number of times reported in site review report, individual interview, and pre-site review forms).

Category	Sub-Theme
Birthing Facility Communication Needs (4/7 reports, 1/3 interviews, 1/7 pre-site review forms)	
	Provide birthing center a report on poor quality specimens
	Inform birthing center when a specimen collected was out of range, and the number of newborns they identified through newborn screening.
	Communicate to whole hospital system rather than just one part
Communication between NBS program and specialists needs to be improved (5/7 reports, 1/3 interviews, 0/7 pre-site review forms)	
	Report out-of-range results to specialists
	Educate clinicians on methods to test for conditions and why changes are made in testing procedures
	Communicate results more efficiently, including using of higher tech solution for reporting results
	Convene meetings between programs and specialists allowing video, phone, in person options for attendance
	Create culture to allow specialists to feel heard/valued
NBS program needs to work with specialists on refining cut-off values (5/7 reports, 0/3 interviews, 0/7 pre-site review forms)	
Needs identified by clinical specialists (4/7 reports, 0/3 interviews, 0/7 pre-site review forms)	
	Develop leadership in lab
	Update ACT sheets and FACT sheets
	Create system for project improvements to increase efficiency of implementation
	Develop age adjusted cut-offs

**Table 3:** Needs identified in State Newborn Screening Program Site Reviews: Birth Provider and Facility Education themes identified (Number of times reported in site review report, individual interview, and pre-site review forms).

Category	Sub-Theme
Need to ensure NBS program’s education materials for birthing providers/birthing center staff are up-to-date (6/7 reports, 3/3 interviews, 0/7 pre-site review forms)	
Need to provide more education/in-service to hospitals (4/7 reports, 1/3 interviews, 1/7 pre-site review forms)	
Needs when Educating Birthing Center Staff	
	Create straightforward trainings by NBS program
	Educate hospitals on best practices around newborn screening (blood spot and point of care screening)
	Develop systems for hospital staff to consistently educate parents
	Ensure hospital staff are aware parents could refuse screen. Some birthing centers have no parent refusals making it likely parents are not given a choice to refuse newborn screen.
	Create system to regularly inform hospital staff about courier pick up logistics for blood spot specimens
	Incorporate newborn screening into annual competencies for hospital staff
	Train hospital staff on collecting dried blood spots to reducing the number of unsatisfactory specimens
Need to educate birthing staff conducting point of care screening on need to screen, how to screen, and need to report data back to the state program (4/7 reports, 1/3 interviews, 1/7 pre-site review forms)	

**Table 4:** Needs identified in State Newborn Screening Program Site Reviews: Information System and Information Technology Needs themes identified (Number of times reported in site review report, individual interview, and pre-site review forms).

Category	Sub-Theme
ST Follow-Up LIMS System/Follow-Up Reporting System Needs (5/7 reports, 1/3 interviews, 2/7 pre-site review forms)	
	Utilize automated reports vs. relying on manual documentation
	Share reports from follow-up with laboratory, including actions taken
	Provide follow-up staff with VPN access for weekend/holiday/after-hours staff
Laboratory LIMS System Needs (5/7 reports, 2/3 interviews, 2/7 pre-site review forms)	
	Identify resources to assist with generating reports
	Develop a system for LIMS to track refusals
	Fully utilize LIMS system and LIMS features
System and/or integrations need to be updated (4/7 reports, 0/3 interviews, 0/7 pre-site review forms)	
LIMS features not designed for laboratory assessments (4/7 reports, 2/3 interviews, 0/7 pre-site review forms)	
Ensure newborns are screened (5/7 reports, 0/3 interviews, 0/7 pre-site review forms)	
	Develop system to track refusals & engage in QA/QC around refusals
	Develop system to track missing specimens and lost to follow-up

**Table 5:** Needs identified in State Newborn Screening Program Site Reviews: Operations in State Newborn Screening Program Needs themes identified (Number of times reported in site review report, individual interview, and pre-site review forms).

Category	Sub-Theme	
Cross-Cutting (Lab and Follow-Up)	Need to update legislation policy/rules (7/7 reports, 0/3 interviews, 0/7 pre-site review forms)	
	Need blood spot storage/retention policy/system (how long store, getting consent to store) (5/7 reports, 0/3 interviews, 0/7 pre-site review forms)	
	Need to assess fee collection/billing process (5/7 reports, 0/3 interviews, 1/7 pre-site review forms)	
	Need to Create or Review Continuity of Operations (COOP) (lab and/or follow-up) (4/7 reports, 1/3 interviews, 0/7 pre-site review forms)	
		- Develop a COOP - Regularly test the COOP
		- Need to involve hospitals, providers, other state, and vendor in COOP practice exercise
Laboratory	Need to update Laboratory Standard Operating Procedures (5/7 reports, 0/3 interviews, 1/7 pre-site review forms)	
		- Update processes and procedures; Include additional processes and procedures needed for special circumstances when appropriate
		- Establish age of specimen when it can be tested
		- Ensure repeat specimen collections occur and are done in a timely manner
		- Communicate when specimen was collected too early or too late to provider
	Need to strengthen blood spot specimen testing process (5/7 reports, 0/3 interviews, 0/7 pre-site review forms)	
	Laboratory Resource Needs (3/7 reports, 1/3 interviews, 3/7 pre-site review forms)	
		- Need additional equipment/software needed
		- Need service contracts on laboratory equipment
		- Acquire additional space / plan for expansion needs
	Hiring and retaining of laboratory staff is difficult (6/7 reports, 2/3 interviews, 6/7 pre-site review forms)	
		- Need more weekend staff (lab and follow-up)
		- Lab staff & follow-up staff not cross-trained so no backup within each piece of the program
		- Lab staff used to do administrative tasks vs purely working on laboratory tasks
		- Lack of opportunity for advancement
Follow-up	Follow-Up Standard Operating Procedures/Protocols (7/7 reports, 0/3 interviews, 4/7 pre-site review forms)	
		- Revise and update follow-up procedures
		- Develop disorder specific protocols for follow-up after notification of positive result
	Need more follow-up staff (4/7 reports, 0/3 interviews, 1/7 pre-site review forms)	
		- Hire for open positions and develop strategy to retain staff
		- Hire staff who are knowledgeable with informatics/IT capabilities to manage laboratory information management systems (LIMS)
		- Hire dedicated QA staff

## Supplementary Materials

The following are available online at http://www.mdpi.com/2409-515X/5/1/13/s1.
