# Supplementary material for: Analyzing Patterns in NewSTEPs Site Review Recommendations: Practical Applications for Newborn Screening Programs
Source: Int J Neonatal Screen. 2019 Feb 12;5(1):13. doi: 10.3390/ijns5010013 (PMC7510203; doi:10.3390/ijns5010013)
Supplement: Supplementary file 1 [file IJNS-05-00013-s001.zip › Online_Supplement_1_newsteps_pre_site_visit_tool.pdf]

A Program of the Association of Public Health Laboratories™

NewSTEPs Pre-Site Visit Tool

1. **Indicate your affiliation with the Newborn Screening (NBS) Program (check all that apply)**

	Lab
	Follow-up
	Other (specify:____)

2. **Self-evaluate how well your state is managing and executing the various components of your NBS program.**

	Very Well	Well	Average	Poor	Very Poor	N/A	Comments
State Legislation and Policy							
Ethics							
Funding Models							
Organizational Structure							
Laboratory System							
Emergency Preparedness							
Short-Term Follow-Up							
Birth Facilities							
Point-of-Care Testing							
Education							
Long-Term Follow-Up System							
Information Technology							
Evaluation							

3. **What would you characterize as the three most significant challenges within your NBS Program? Please provide as much information as you can so we can prepare for the visit.**

4. **How well do the different parts of your NBS systems communicate?**

	Very Well	Well	Average	Poor	Very Poor
Hospital(s) and the Lab					
Lab and Follow-Up					
Follow-Up and the Hospital(s)					
Follow up and Specialist(s)					

   a. **How well is your NBS laboratory and follow up program integrated? (place a check next to the description that best fits)**

	They are completely integrated
	They work well together but there is room for improvement
	They are somewhat integrated but do miss things occasionally
	They are not well integrated and often things get missed
	They are not at all integrated

5. **What are your most significant accomplishments in the last 2-3 years??**

6. **What would you like to get out of a site visit? What are your needs?**

7. **Are there any specific questions or issues that you would like the site visit team to focus on and address? Please prioritize these for the team and identify specific questions that need to be addressed. Please note: the answers below will help us to customize our visit to your site, but we will not limit ourselves to only these components during the comprehensive visit.**

8. **When was your laboratory's last Clinical Laboratory Improvement Amendments (CLIA)/ College of American Pathologists (CAP) inspection?**

9. **We are interested in obtaining the following materials if you have them readily available prior to site visit as well as anything else you think may be of value to the team.**

- NBS Collection Form
- Quality Assurance Plan
- Organizational chart for NBS program
- Provider educational materials—Practitioner's Manuals, fact sheets, websites, etc.
- Parent educational manuals –parent brochures in particular
- Laboratory and Follow-up algorithms
- Examples of Lab Reports (normal and abnormal for each screening category)
- Consent/dissent policy for NBS
- Consent/dissent policy for residual Dried Blood Spots (DBS)
- Release of Information Policy
- Laws/statutes
- List of local hospitals or number of birthing hospitals with annual birth rate
- Annual number of births for the state
- List of specialists
- Website(s)
