# Supplementary material for: Analyzing Patterns in NewSTEPs Site Review Recommendations: Practical Applications for Newborn Screening Programs
Source: Int J Neonatal Screen. 2019 Feb 12;5(1):13. doi: 10.3390/ijns5010013 (PMC7510203; doi:10.3390/ijns5010013)
Supplement: Supplementary file 1 [file IJNS-05-00013-s001.zip › Online_Supplement_3_NewSTEPsSiteReview_FollowUpSurvey.pdf]

NewSTEPs

A Program of the Association of Public Health Laboratories™

It has been over 6 months since the NewSTEPs Site Review Team visited your state. We would like to know how helpful the process and report were for you so that we can make changes to our process, if needed. We would also like to know if you would more support from NewSTEPs. Please take a few minutes and answer the following the questions for us.

1. Did you find the site review report helpful? ____ yes _____ no
   a. If yes, how was it helpful?
   b. If no, what, in your view, can be done to make the report more helpful or useful to a state?

2. What else do you need from NewSTEPs to help you make changes you would like to make?

3. What resources or connections, if any, could NewSTEPS make that might be helpful for your state?

4. Have you implemented any of the recommendations within the report?
   a. If so, what have you done or started to address?
   b. How have you started to address this?
   c. Can you identify any barriers to implementing recommendations in the report?

5. Is there anything significant you have done in your program that you want to share with us?

6. Is there a change you would like to make but are having difficulty? If yes, what?
   a. Can we talk to you about how NewSTEPs can help you? (if yes provide a name and contact number)

Name: ____

Contact number:____
