# Supplementary material for: Analyzing Patterns in NewSTEPs Site Review Recommendations: Practical Applications for Newborn Screening Programs
Source: Int J Neonatal Screen. 2019 Feb 12;5(1):13. doi: 10.3390/ijns5010013 (PMC7510203; doi:10.3390/ijns5010013)
Supplement: Supplementary file 1 [file IJNS-05-00013-s001.zip › Online_Supplement_2_newsteps_site_review_manual_version_6.pdf]

NewSTEPs Site Review Manual – No Space

Version 6
for the

NAME OF PROGRAM

Newborn Screening Program

Last Updated: July 1, 2016

Name of Reviewer: ____________________________

Expertise on the team:
- o Laboratory
- o Follow-Up
- o Hospital
- o Health Information Technology
- o Medical Provider/Specialist
- o Diagnostic Testing
- o Other: ________________________

*This version has no spaces for notes.

Table of Contents

NewSTEPs Site Review Manual – Less Space 1
Acknowledgements 4
Disclaimer 5
Background 5
Facilitation Tips 6
Section 1: Program Overview 7
Initial Program Overview Summary of Key Points 8
State Legislation and Policy 9
Funding Models 10
Organizational Structure 11
Emergency Preparedness 12
Section 2: Continuous Quality Improvement 13
Continuous Quality Improvement Summary of Key Points 13
Continuous Quality Improvement Plan 13
Conducting a Self-Evaluation 13
Use of Evaluation Information 13
Section 3: Laboratory System 14
Laboratory Summary of Key Points 15
Checklist of Documents to be reviewed by Site Review Team (SRT): 14
Discussion with Laboratory Staff and/or Supervisors 16
Laboratory Staffing and Procedures 16
HIT/LIMS System 16
Ethics as related to laboratory procedures, storage, and destruction 17
Tracking and reporting timeliness 18
Laboratory Tour 18
Delivery of blood spot samples to the lab 18
Recommendations from Laboratory Staff 18
Section 4: Follow-Up 19
Short-Term Follow-up Overview Summary of Key Points 19
Checklist of follow-up documents to be reviewed by Site Review Team (SRT): 19
Follow-Up Staffing and Procedures 21
Long-Term Follow-Up System 21
HIT/LIMS/Information System 22
Ethics – Follow Up System 22
Meeting with Follow-Up staff Without Supervisor 22
Section 5: Point of Care Testing 23
Checklist of Documents to be reviewed by Site Review Team (SRT): 23
Hearing (EHDI) 24
Critical Congenital Heart Disease (CCHD) 25
Section 6: Education (Follow-Up, Birthing Centers, Laboratory, and Specialists) 27
Checklist of follow-up documents to be reviewed by Site Review Team (SRT): 27

Education for Parents 28
Prenatal Education for Parents 28
Education for Providers 28
Education for Birthing Centers 29
Education for Midwives 29
Recommendations around Education 30
Section 7: Birth Facilities 31
Checklist of follow-up documents to be reviewed by Site Review Team (SRT): 31
Birthing Facility Summary of Key Points 32
Birthing Facility #1 33
Birthing Facility #2 33
Sit Down Meeting with Birthing Facility 33
Questions for Well-Baby Nursery Birthing Facility 34
Questions for the NICU Birthing Facility 35
Questions for Hospital Lab Birthing Facility 35
Section 8: Medical Providers/Specialists 36
Section 9: Information System 37
Information System Summary of Key Points 37
Overview of State's HIT Systems 38
Perceived Utility of Information System 38
Use of Data 38
Recommendations around the Information System(s) 40
Section 10: Next Steps 41

Acknowledgements

The development of this Newborn Screening Technical assistance and Evaluation Program (NewSTEPs) Site Review Manual was supported by Cooperative Agreement #U22MC24078 from the Maternal and Child Health Branch (MCHB) of the Health Resources and Services Administration (HRSA). Its contents are solely the responsibility of the authors and do not necessarily represent the official views of HRSA.

We thank the NewSTEPs Steering Committee and the NewSTEPs Evaluation Workgroup for the contribution of their time and expertise to provide substantial guidance on the development of this tool. The NewSTEPs Evaluation Workgroup was comprised of the following individuals:

- Harry Hannon, PhD, retired, Centers for Disease Control and Prevention
- Patrick Hopkins, BS, Chief Newborn Screening Unit, Missouri State Public Health Laboratory
- Elizabeth Jones, MPH, Senior Specialist, Newborn Screening and Genetics Program, Association of Public Health Laboratories at the time this was created.
- Yvonne Kellar-Guenther, PhD, NewSTEPs Evaluator, Colorado School of Public Health
- Julie Luedtke, BS, Newborn Screening Program, Nebraska Department of Health and Human Services
- Jelili Ojodu, MPH, Director, Newborn Screening and Genetics Program, Association of Public Health Laboratories
- Susan Tanksley, PhD, Laboratory Operations Unit Manager, Laboratory Services, Texas Department of State Health Services
- Judi Tuerck, MS, RN, retired, Oregon Health & Science University
- Thalia Wood, MPH, Specialist, NewSTEPs, Association of Public Health Laboratories

Many pre-existing documents served as references for the development of the NewSTEPs Site Review Manual including the Clinical and Laboratory and Standards Institutes (CLSI) Documents (*LA4-A-5 Blood Collection on Filter Paper for Newborn Screening Programs, I/LA27-A Newborn Screening Follow-Up, I/LA35-A Newborn Screening for Cystic Fibrosis, I/LA31-A Newborn Screening for Preterm, Low Birthweight, and Sick Infants, I/LA32-A Newborn Screening by Tandem Mass Spectrometry*) and the Performance and Evaluation Assessment Scheme (PEAS) developed by HRSA and the National Newborn Screening and Genetics Resources Center (NNSGRC). Additionally, results from a Delphi survey fielded to state newborn screening (NBS) programs and Quality Indicators developed by the NBS community in partnership with NewSTEPs contributed to the development of this tool.

Disclaimer

NewSTEPs Site Visit Review Tool is a dynamic document. It will be updated routinely by the NewSTEPs staff, with input from the newborn screening (NBS) community.

Background

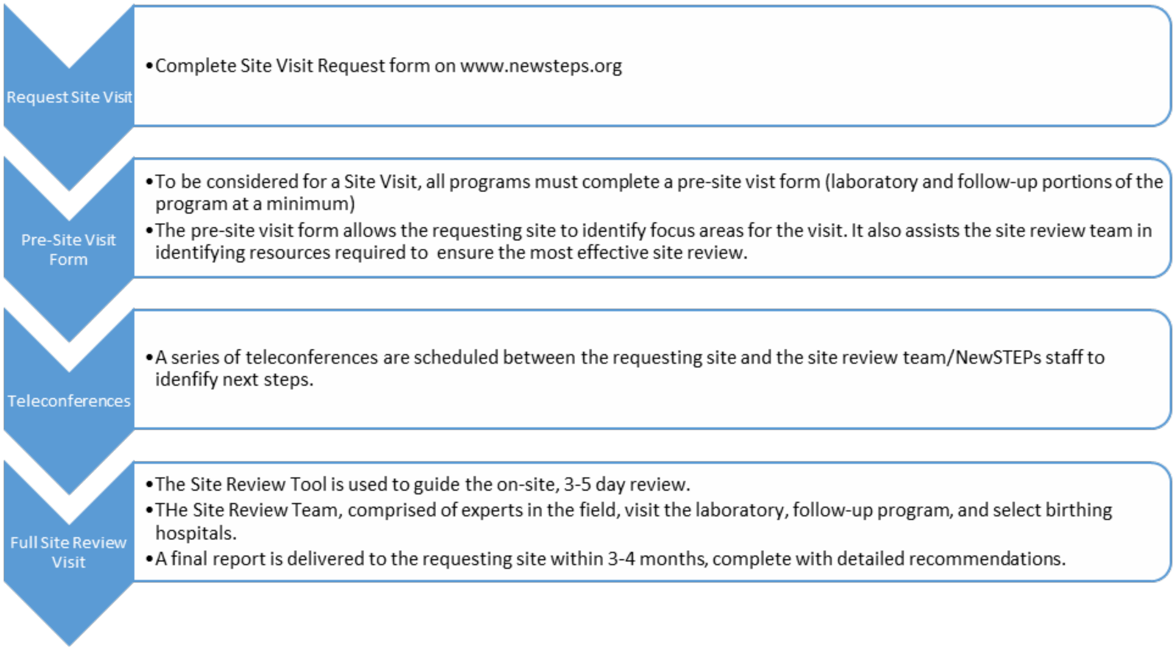

As a means to achieve quality improvement in the NBS system, NewSTEPs conducts comprehensive site visits to NBS programs, upon their request, to assess resources, methods, and procedures. These comprehensive site visits include a review of all aspects of the NBS system from collection at the hospital, laboratory analysis, education, treatment, and follow-up components. The visits are designed to provide constructive peer-to-peer feedback to the program to highlight areas of strengths and areas of need. The visits are neither regulatory nor punitive. A team of five to six experts representing various areas of the NBS system conduct the comprehensive visits, reviewing all components of the NBS program over the course of several days to a week. Each visit is customized to reflect the needs of each program.

This manual reviews large programmatic areas including state legislation and policy, ethics, funding models, organizational structure, point of care testing, education, and more.

Upon completion of the NewSTEPs Site Review Visit, the NBS program will receive oral feedback as well as a comprehensive written report. Technical assistance is a primary mission of NewSTEPs and continues well after the Site Review Visit is completed.

Facilitation Tips

Below is a list of different types of personalities we may encounter in group discussions and some tips on how to ensure everyone has the opportunity for their voice to be heard.[1]

1) "The Superstar" *is the participant who knows all and tells all, chiming in at every opportunity during the meeting. They offer good information, but their presence is dominating and potentially biasing the group.*

Moderator Tip: Tell them you appreciate their thoughts and that the feedback they have provided to this point is valuable; however you would also like to hear from other **focus group** participants.

2) "The Introvert" *is the participant that is generally quiet and non-opinionated in fear of others disagreeing with their thoughts. When the Introvert does finally get around to offering an opinion and another participant disagrees, the Introvert shuts down completely.*

Moderator Tip: Make it very clear in the beginning of the group that there is no right or wrong answer. Encourage this participant to voice their opinion no matter what, get them involved, if you see them agreeing or disagreeing with another's comments (nodding their head/shaking their head), follow-up to them.

3) "The One Shining Moment Participant" *is a relatively quiet participant who tends to agree or disagree with the majority. It's the participant who offers nothing more than a "yeah, it's okay" or "no, I don't like it." Then, all of a sudden this participant gives the group a golden nugget of information. After that, the participant reverts to the depths of normality.*

Moderator Tip: Get this person involved early and often. There is most likely more that he/she has to offer. If the moment doesn't happen until the tail-end of the group, make sure you follow-up and expand on the comment with the participant before the time has passed.

[1] This text comes from George K.'s online blog entitled *Focus Group Moderator Tips to Handle 7 Unique Personalities.* The information as accessed on 6-28-16 from https://rmsbunkerblog.wordpress.com/2010/08/20/focus-group-moderator-tips-to-handle-7-unique-personalities-focus-group-facility-in-watertown-ny-northern-ny/.

Section 1: Program Overview

This section includes:

- ✓ A list of what should be included in the Program Overview Presentation
- ✓ Questions about:
  - State Legislation and Policy
  - Advisory Committee
  - Funding Model
  - Organizational Structure
  - Emergency Preparedness

Initial Program Overview Summary of Key Points

Goal: The Site Review Team (SRT) receives an overview of the program including information on state legislation and policy, the advisory committee, the funding model, the organizational structure (NBS within the health department and how lab and follow-up work together), and emergency preparedness.

At the initial meeting, the host site staff should be required to provide a PowerPoint presentation for the team that at minimum covers:

1- Overview of the volume
   a. # of specimens tested per year
   b. # of birthing hospitals/centers
   c. # of out of hospital providers/% of births that are out of hospitals
2- An overview of how the program is structured including:
   a. state legislative action and rules,
   b. organizational structure,
   c. overview of the lab program,
   d. overview of point of care testing,
   e. overview of the follow-up program, and
   f. funding models.
3- An overview of the newborn screening process (from getting a sample to communicating the result, from hearing a result to contact with the family) including the timing of the activities.
4- An explanation of how the state engages in Continuous Quality Control/Quality Assurance/Quality Improvement.
5- A list of concerns the state wants to make sure the Site Review Team (SRT) addresses.
6- A description or explanation of what is the state's key strength.

NOTE to SRT: If you do not hear these things, please ask questions about them.

The team may want to decide if they need a lead for this section.

The goal is for you to understand the structure of the program and begin to identify strengths and challenges that you can further probe when you talk to the laboratory, follow-up, hospital, and specialists later during the visit.

State Legislation and Policy

You want to understand statute, law, rules or regulation covering Newborn Screening (NBS) (for bloodspot screening), what is covered by these, how it is changed, etc.

1- How does the program ensure it is meeting the statute/law/rule/regulation? How often is that done?
2- Who is responsible for reviewing the statute/law/rule/regulation?
3- What is covered by statute/law/rule/regulation?

Areas of Statute/Law/Rule/Regulation
NBS program responsibilities
Parental consent/dissent
Specimen collection time after birth
Transport of specimen to the lab (including time limits, type of courier service, etc.)
Testing
Reporting
Short-term follow up (confirmation testing)
Long-term follow up
Early discharge
Hospital transfers
Out of hospital births
Neonatal Intensive Care Unit (NICU) guidelines
Procedure for adding tests

State Legislation and Policy Highlights

Challenges/Barriers/Concerns
Strengths

Advisory Committee

Your goal is to understand the make-up and authority of the advisory committee:
1- Tell us about your advisory committee.
Make sure the following is covered:

a. roles/positions/organizations are represented on committee? (listen to see if laypersons are included on this list)
b. how NBS advisory committee utilized?
c. Rules/regulations for advisory committees?
d. What authority does the NBS advisory committee have?

Advisory Committee Highlights

Challenges/Barriers/Concerns
Strengths

Funding Models

Your goal is to understand the how much the fee is, what is done with the fee, how the fee is changed, what happens to the fee, what other money is used to fund NBS?

1- What is the program's fee structure? How much is the fee?
   a. First screen, second screen, repeat screens.
   b. Is the fee for the second screen part of the first screen?

Areas from which NBS is paid
General Funds
NBS Fee
Licensing Fee
Other (specify)

2- How is the fee collected?

3- What do the fees pay for?

Activities Paid for by NBS fees
Lab tests (To include salaries of laboratory personnel, supplies, instruments and equipment maintenance)
Long term follow-up support (to include physician consultants, geneticists, genetic counselors, etc.)
Critical Congenital Heart Disease (CCHD) services
Early Hearing Detection and Intervention (EHDI) services
Administration (space rental, human resources support, fiscal services, travel, etc.)
Courier services
Information Technology (IT) support (including Laboratory Information Management Systems (LIMS), networking support, hardware support, etc.)
Development/support fund

4- How are NBS fees collected?
   Purchase collection kits?
   Bill hospitals/submitters?
   Bill Medicaid/Insurance?
   Other?

5- What happens with the fee?
   - Revert to state general fund
   - Program keeps for operating expenses
   - Other (explain)

6- Any challenges with billing?

7- When would fees be waived?

8- How are fees increased?

9- Who is responsible?

Funding Model Highlights

Challenges/Barriers/Concerns & Strengths

Organizational Structure

Look at Organizational Charts

Your goal is to understand:

- the reporting and management structure of the NBS program, lab, and follow-up
- how NBS fits within the health dept. and how valued this program is or is not.
- The relationship between lab and follow-up
- Who is evaluates the performance of the assays/tests
- Who calls on unsat specimens and borderline results

1- How does NBS fit into the mission and vision of your public health department?

2- Does the NBS program feel the NBS system is valued by the state's public health leadership?

What are the strengths or problems with the reporting structure?

3-How do the lab and follow-up work together?

a. What leads to a good or poor working relationship? (location, in same dept/division/reporting structure, day-to-day interaction)
b. How often do the programs meet?
c. How are concerns communicated?
d. If the laboratory and follow-up programs are not physically unified, how connected are they (i.e., shared databases, cross-trained staff, etc.?)

4- Ask staff what change they would make to the lab and follow-up relationship.

Listen to see how well current organizational structure (relationship between laboratory and follow-up) is working?

a. What are the strengths, weaknesses, opportunities and threats associated with this structure?

5-Who evaluates the performance of the assays/tests [Positive Predictive Value (PPV), Negative Predictive Value (NPV), False Negative (FN), False Positive (FP)]?

6-Who makes the follow-up calls on unsatisfactory specimens?

7-Who makes the follow-up calls on borderline results?

Organizational Structure Highlights

Challenges/Barriers/Concerns

Strengths

Emergency Preparedness

Your goal is to understand if the program has an emergency preparedness plan, do they have an agreement with another NBS program, and if they have what they need in place.

1- Explain your emergency preparedness plan (Continuity of Operations Plan (COOP)).
   a. What does it cover? (storage, shipping samples, reporting results, resuming services by home lab)
   b. Have you had to use it?
      i. If so, how did it work? What challenges did you face? What worked well?
   c. Ask about how the plan is updated. (who updates, how often, when last updated?)
   d. Do you TEST the plan? If yes, when was the last time?
      i. What part/pieces did you practice?

2- Do you have any concerns about your plan?

3- What is your policy or agreement, for collaborating with other NBS programs to provide support to a program in case of disaster recovery?
   a. Which other state(s) support you? What is covered?
   b. Which other states do you support? What do you do for them?

   You can ask to see a copy of the plan.
   Find out if they have used and/or tested these plans.

4- What emergency preparedness activities has the state done (e.g., table top exercises, specimen exchange drills)?

5- Have you ever had to rely on your COOP in a real-life emergency? If yes, how did it go?

Emergency Preparedness Highlights

Challenges/Barriers/Concerns
Strengths

Section 2: Continuous Quality Improvement

This section includes:

- ✓ Questions about:
    - A Continuous Quality Improvement Plan
    - Conducting a Self-Evaluation
    - Use of Evaluation Information

These questions can be covered at any time during the site visit. Ideally management in the lab and follow-up should be present to discuss the CQI approach the newborn screening program is taking. If the program has a CQI lead, that person should also be present. If time is tight, this entire section may not be covered.

The goal is to determine if how the program continues to monitor their progress and improve their system.

The group should decide who is most comfortable asking these questions.

Continuous Quality Improvement Summary of Key Points

At the end of this section, the team should be able to address the following:

- If the program engages regularly in CQI
- If there a CQI plan
- How the program ensures the findings from their CQI are used

Continuous Quality Improvement Plan

1- What is your continuous quality improvement plan?
2- What is your biggest barrier to engaging in CQI?

Conducting a Self-Evaluation

1- Have you conducted a self-evaluation? If yes, how often do you do this? When was the last one?
   If no, do you have plans/resource for self-evaluation?
      a. Do you want assistance?
2- Would you be willing to participate in regional evaluations?

Use of Evaluation Information

1- Tell us about any your most recent CQI activities you have done/have conducted.
2- How have those findings been used by your program?

Continuous Quality Improvement Highlights

Challenges/Barriers/Concerns
Strengths

Section 3: Laboratory System

To fill out this section you should:

- Tour the lab
  - From receiving to testing to report out
- Talk to lab staff about flow, staffing, what is working well, what changes they would like to see, what challenges they face
- Look at SOPs and training materials

The lab expert(s) should be the lead but the rest of the team should also feel free to ask questions. The HIT expert should also probe on the IT, HIT, and LIMS systems.

The goal is to understand the lab workflow, how they engage in quality assurance and continuous quality improvement, how they support staff and minimize errors.

You also want to understand the HIT system and ethics around lab procedures, storage and destruction.

This section includes questions about:

- Lab hours and staffing
- Delivery of blood spot samples to the lab
- Tracking and reporting timeliness
- HIT System
- Ethics as related to laboratory procedures, storage, and destruction

Checklist of Documents to be reviewed by Site Review Team (SRT):

- [ ] The SOPs
- [ ] Internal audit documents
- [ ] Quality Control Manuals

Notes on documents:

Laboratory Summary of Key Points

At end of meeting with the lab staff and taking a tour you should be able to address if the lab has:

- Sufficient personnel
- Adequate training for staff
- Training protocols
- Cross-trained personnel
- Clearly defined roles
- Written procedures and policies
- Adequate equipment/instruments
- Cutoffs that work well?
  - Process for adjusting cutoffs that works well?
- Algorithms
- Easy to understand workflow
- Quality Assurance (QA)/Quality Control (QC) manuals/QA Plan
  - Participation in the Centers for Disease Control and Prevention Proficiency Testing/QC programs

- Knows the false positive/negative rates
- A method to track timeliness (e.g. time stamps)
- Adequate timeliness (birth to collection, birth to receipt, collection to receipt, birth to report out, receipt to report out)
- A method for reporting timeliness to hospitals
- Adequate method for reporting out unsatisfactory specimens
- Adequate procedure for getting repeat specimens
- Reasonable unsatisfactory rate
- A method for reporting prevalence of disorders
- A method for collecting missing essential information from bloodspot card
- A method for reporting infants not receiving valid screenings due to death, parental refusal, error or missing 2nd screen
- Adequate IT/LIMS/HIT
- A comprehensive, easy-to-interpret laboratory report
- How well Lab and Follow-Up work together

Discussion with Laboratory Staff and/or Supervisors

Laboratory Staffing and Procedures

[might be easier in a meeting before or after tour]

1- How many days does the lab operate?
   a. What happens on weekends/holidays?
      - Receiving specimens
      - Running tests
      - Running repeat tests to confirm out of range results
      - Calling out all out of range results
      - Other, please specify
   b. If not open six (6) days a week or more are they trying to change that? What are barriers? What support need to make change?

2- Please provide an overview of your laboratory staffing.
   a. Do they have adequate staff? Too many? Too few?
   b. How cross-train staff?
   c. Suggestions to improve staffing?

3- What is your relationship with follow-up like?
   a. How are you trained on what they do?
   b. What is working well?
   c. What is one change you'd like to make?

HIT/LIMS System

1- Tell us about your current HIT System.
   a. Is it meeting the needs of the lab?
   b. How do you share information with follow-up?

Ethics as related to laboratory procedures, storage, and destruction

1- How are parent refusals for screening tracked and documented?
   a. Is there any additional contact with families who refuse screening from the program?

2- What is your program's policy on the ***use*** of residual Dried Blood Spots (DBS) after NBS is completed? (i.e., use in new assay development, use in research)
   a. Is it a written policy? Has it been reviewed by a legal entity?
   b. Is there a law or statute to support this written policy on the use of residual Dried Blood Spots (DBS) after NBS is completed?

3-What is your program's policy on the ***storage*** of residual DBS after NBS is completed?
   a- Is it a written policy? Has it been reviewed by a legal entity?
   b- Is there a law or statute to support this policy?

4-What is your program's policy for the ***destruction*** of residual DBS after NBS is completed?
   a. Is it a written policy? Has it been reviewed by legal entity?
   b. Is there a law or statute to support this policy?
   c. How are parent/guardians notified of residual DBS storage and use?

5-Does residual DBS storage and use require parental consent, or can parents opt out?

6-How does your program handle incidental findings revealed from NBS?
   a. carrier status?
   b. maternal genetic conditions?
   c. variants of unknown significance?
   d. other?

Tracking and reporting timeliness

1- What is the recommended time period for samples to be received by the NBS laboratory?
    a. Is this a state policy/practice/recommendation/law?
2- How do you handle birthing centers or out of hospital birthing providers who do not comply?
3- How does your state track timeliness of specimen delivery to the laboratory?
    a. How track timeliness of specimen delivery, identify issues, and create a plan to address those issues?

  [listen for:]

  NBS program keep a record of transit performance by hospital?
  NBS program review the transit performance times?
  NBS provide feedback to birthing hospitals regarding transit times?
4- How do you give feedback to hospitals and midwives regarding the timing of specimen receipt by the NBS laboratory? (report cards, education materials, Quality Improvement tools) How often?
5- What are the program's challenges to reporting out the critical results with 5 days of life and all results within 7 days of life? (ACHDNC recommendations).

Laboratory Tour

Delivery of blood spot samples to the lab

1- Explain your courier system (i.e., FedEx, UPS, Private Service, etc.). Who is it? How often pick up? Which hospitals have them?
    a. Look over the policy for courier service.
    b. Is courier required for all birth hospitals? Who pays for service (hospital, program, other)?
2- How is the delivery of NBS samples/specimens to the NBS laboratory paid for?
3- How do the majority of specimens get from birthing hospitals to the NBS laboratory? For home births?
    - Courier services
    - Overnight services
    - U.S. Mail
    - Other

Recommendations from Laboratory Staff

1- If you could make 1 change to make your job easier, what would it be? [ask all staff]

Laboratory Highlights

Challenges/Barriers/Concerns & Strengths

Section 4: Follow-Up

You may also need to cover Point of Care Testing, and Education with this group.

To fill out this section you should talk to follow-up staff with and without the supervisor(s) present. This is often a discussion in a conference room but, if possible, should include conversations as you tour their workspace. This section includes:

- ✓ Questions about:
  - Short-Term Follow-Up System
    - Questions to ask the STFU staff without the supervisor present
  - Long-Term Follow-Up System
  - Ethics as related to follow-up

The follow-up experts should be the lead but the rest of the team should also feel free to ask questions. The HIT expert should also probe on the IT, HIT, and LIMS systems.

The goal is to understand the follow-up workflow, how they engage in quality assurance and continuous quality improvement, how they support staff and minimize errors.

You also want to understand the HIT system and ethics around parental refusals and identification of sensitive information (e.g. parentage)

Checklist of follow-up documents to be reviewed by Site Review Team (SRT):

- ☐ The SOPs
- ☐ Internal audit documents
- ☐ Quality Control Manuals

Notes on follow-up documents:

Short-Term Follow-up Overview Summary of Key Points

At end of talking with follow-up staff you should be able to address if Follow-Up has the following:

- Sufficient personnel
- Adequate training for staff
- Training protocols
- Cross-trained personnel
- Clearly defined roles
- Written procedures and policies – for STFU and LTFU
- Protocol to communicate with providers
- Protocol to communicate with families
- Easy to understand workflow
- Algorithms
- Quality Assurance/Quality Control reports
- Method of tracking lost-to-follow-up
- Reasonable lost-to-follow up rate
- Way to track timeliness from collection to diagnosis
- Adequate IT/LIMS/HIT
- How well Lab and Follow-Up work together
- Adequate EHDI screening and surveillance program (Section 5)
- Adequate CCHD screening and surveillance program (Section 5)
- An adequate plan for educating parents, pregnant mothers, primary care providers, birthing center staff, and midwives (Section 6) including:
    - Adequate education tools with appropriate messaging
    - Adequate dissemination techniques
    - Adequate plan to determine effectiveness and need for change

Follow-Up Staffing and Procedures

1- Can you go over the staffing of follow-up? What is everyone's role?
   a. Listen to see if they have adequate staff? Too many? Too few?
   b. How cross-train follow-up staff?
   c. Suggestions to improve staffing?
2- Do sub specialists serve as medical consultants to the program?
   a. If yes, what is their role [listen to see if follow-up is part of their role], and are they paid by your program?
3- What are the NBS follow-up program hours of operation?
4- How do you handle weekends?
   a. What is done? By whom? What do you call out?
   b. Where do staff work?
5- Does your NBS follow-up program have after hours paging/on call services? How does that work? When do you use this?
6- What is your relationship with the lab like?
   a. How are you trained on what they do?
   b. What is working well?
   c. What is one change you'd like to make?
7- How do you track if your program receives confirmatory testing for all babies for blood spots?
   a. How does it work for you?
   b. How flexible are they [the lab, LIMS] in making changes for you?
   c. Is it meeting your needs?
8- Describe the process you use when your program identifies a baby who did not receive a newborn screening?
9- How do you determine when a baby is lost to follow-up?
   a. How do you close a case?

10- How do you deal with families who do not want to travel for testing?

Long-Term Follow-Up System

1- What long-term follow-up data do you collect on each patient?
2- Who collects the long-term follow-up data (which department/section/division)?
   a. How is it utilized?
   b. What are the challenges in getting long-term follow-up data?
3- How long do you follow the babies/get long term follow-up data?
4- How do you track if every affected infant and family has access to specialized treatment center?
5- Describe your care coordination plan?
6- What are the resources and/or laws that help to fund medical foods/treatment in your state?

HIT/LIMS/Information System

1- Tell me about your information system. What are you using?
    a. Does it meet the needs of follow-up?
2- How do you share information about cases with the lab?

Ethics – Follow Up System

1- How are parent refusals tracked and documented?
2- What additional contact, if any, is there with families who refuse screening?
3- Describe your state's genetic privacy regulations (if applicable).
4- How does your program handle unintended information that is revealed from NBS (e.g., carrier status, paternity, maternal genetic conditions, variants, etc.)?

Meeting with Follow-Up staff Without Supervisor:

1- How do you feel things are going?
2- If they have nothing to say, ask them to walk you through a case (fictitious or real). How was it handled? What worked well on this case? What didn't work well on this case? What would you change if you were the supervisor?
3- What is one change you'd like to see that will help you do your job better or make your life easier?

Follow-Up Highlights

Challenges/Barriers/Concerns
Strengths

Section 5: Point of Care Testing

To fill out this section you should talk to follow-up staff. This section includes:

- ✓ Questions about:
  - Early Hearing Detection and Intervention (EHDI)
  - Critical Congenital Heart Disease

You will also learn about these tests when you talk to birthing center staff.

The follow-up experts should be the lead but the rest of the team should also feel free to ask questions.

The goal is to understand who is conducting these tests, what data is collected and provided to the newborn screening program, how abnormal tests are handled, and how confirmed cases are handled.

Checklist of Documents to be reviewed by Site Review Team (SRT):

- ☐ The SOPs
- ☐ Any education to birthing centers on how to conduct testing
- ☐ Any education to birthing centers on interpreting results of the tests
- ☐ Data collection tools or any materials showing how the data is stored

Hearing (EHDI)

1- Who in the state has program oversight for EHDI?
2- Does your state have a legislative mandate for newborn hearing screening?
    a. If no, is newborn hearing screening offered to all babies in the state?
3- Who does the EHDI screening? What problems, if any, do you have getting this test conducted?
4- Are you able to track if your program receives confirmatory testing for all babies for EHDI? If yes, how is it recorded in your records/LIMS?
    a. How does it work for you?
    b. Is it meeting your needs?

5- What type of data do you get at the public health level for babies with abnormal hearing screen?

✓	Method
	Date and time of follow-up evaluation for positive screens, and name of specialist.
	Date and time of follow-up evaluation for positive screens, name of specialist, and final diagnosis.
	Date and time of follow-up evaluation for positive screens, name of specialist, final diagnosis, and long-term follow-up data.
	Positive screens had follow-up evaluation indicated by a Yes or No.
	Not clear what data is or will be collected at the public health level.

6- What type of data do you collect regarding the infants with a diagnosis of a hearing loss? [PROBE for:]
    - Type of diagnostic work up
    - Details of work up
    - Modalities for hearing if there is a loss of hearing
    a. When and how is the data collected?

✓	Method
	Electronic Birth Certificate
	Electronic system (e.g. OZ or other data transfer system)
	Paper forms
	Blood spot cards
	Other: (list)
	Not collecting numeric data – just pass/not pass
	Numeric data

7- What type of data do you receive on the work up done for positive screens?
8- Who reviews the data to ensure the quality of the EHDI screening program?
9- How does your programs document what kind of technology is used for heag screening within the state?

EHDI Highlights

Challenges/Barriers/Concerns & Strengths

Critical Congenital Heart Disease (CCHD)

1- Who in the state has program oversight for Critical Congenital Heart Disease CCHD?
2- Does your state have a legislative mandate for newborn screening for critical congenital heart disease?
    a. If no, is CCHD screening offered to all babies in the state?

3- Who does the CCHD screening? What problems, if any, do you have getting this test conducted?
4- Are you able to track if your program receives confirmatory testing for all babies for CCHD? If yes, how is it recorded in your records/LIMS?
    a. How does it work for you?
    b. How flexible are they [the lab, LIMS] in making changes for you?
    c. Is it meeting your needs?

5. Is the state health department collecting data for CCHD?
    a. If yes, who collects the screening results (which program department/division)?
    b. Use the charts below to indicate where the information is recorded and what type of data is collected.

✓	Method
	Electronic Birth Certificate
	Electronic system (e.g. OZ or other data transfer system)
	Paper forms
	Blood spot cards
	Birth defects registry
	Other: (specify)

Type of Data	For Whom?
Collect all oxygen saturations and times	o All babies<br>o Babies who fail<br>o Babies with Diagnosis
Collect final oxygen saturation (and test #) only	o All babies<br>o Babies who fail<br>o Babies with Diagnosis
Collect pass/fail/not done	o All babies<br>o Babies who fail<br>o Babies with Diagnosis
Long-term follow-up outcome data	o All babies<br>o Babies who fail<br>o Babies with Diagnosis

6-Do you receive data on the work up completed for positive screens?
7-What type of data do you receive on the work up done for positive screens?
[you might hear the following]

- Echo
- Physical Exam
- Chest x-rays

8-Who reviews the data to ensure the quality of the CCHD screening program?

9-How does your programs document what kind of technology is used for CCHD screening within the state?

CCHD Highlights

Challenges/Barriers/Concerns
Strengths

Section 6: Education (Follow-Up, Birthing Centers, Laboratory, and Specialists)

To fill out this section you should talk to follow-up staff. This section includes:

- ✓ Questions about:
  - Education for Parents
  - Education for Providers
  - Education for Hospitals

You will also learn about education when you talk to birthing center staff.

The follow-up experts should be the lead but the rest of the team should also feel free to ask questions.

The goal is to understand how the program is educating parents, providers, birthing centers, and midwives. This not only includes what education messages are created but how these messages are created and how they are disseminated.

Checklist of follow-up documents to be reviewed by Site Review Team (SRT):

- ☐ Education materials for parents
- ☐ Education materials for pregnant moms
- ☐ Education materials for PCPs/midwives
- ☐ Education n materials for birthing centers

Education for Parents

1- What is your role in educating parents about newborn screening?
2- Who else do you work with to facilitate and deliver newborn screening education to parents?
3- How are parents educated about newborn screening?
    a. What are the focus of the message (e.g. importance of newborn screening, need for second screen)
4- How do parents get this educational information?
5- How do you monitor if this education is being delivered to parents?
6- How do you know if the education is working?
7- How often do you review of the parent education plan? How do you do the review?
    a. How do you decide if you need to make changes or update the plan/materials?

Prenatal Education for Parents

1- What are the prenatal educational materials for expecting parents?
2- How are these given to parents (online, social media, brochures, etc., at Ob office, birthing center)?
3- How often are they updated? When they were last updated?

Education for Parents Highlights

Challenges/Barriers/Concerns
Strengths

Education for Providers

1- What is your role in educating providers about newborn screening?
2- How are providers educated about newborn screening?
    a. What are the focus of the message (e.g. importance of newborn screening, need to get parents in quickly for retests)?
3- How do providers get this educational information?
4- How do you monitor if this education is being delivered to providers?
5- How do you know if the education is working?
6- How often do you review of the provider education plan? How do you do the review?
    a. How do you decide if you need to make changes or update the plan/materials?

Education for Providers Highlights

Challenges/Barriers/Concerns
Strengths

Education for Birthing Centers

1- What is your role in educating hospital staff about newborn screening?
2- Who else do you work with to facilitate and deliver newborn screening education to hospital staff?
3- How are hospital staff educated about newborn screening?
    a. What are the focus of the message (e.g. importance of newborn screening, need for second screen)
    b. Which staff do you target? (Well-baby, NICU, lab)
    c. If more than one group, how do you adjust the education for the different roles?
4- How do you monitor if this education is being delivered to all the hospital staff who touch a newborn screening collection kit?

5- How do you know if the education is working?
6- How often do you review of the hospital education plan? How do you do the review?
    a. How do you decide if you need to make changes or update the plan/materials?

Education for Midwives

1- Tell us about the midwives you have in your state (certified, noncertified (potentially illegally practicing), lay midwives, various religious groups).
2- What is your role in educating all midwives in your state about newborn screening?
3- Who else do you work with to facilitate and deliver newborn screening education to midwives?
4- How are midwives educated about newborn screening?
    a. What are the focus of the message (e.g. importance of newborn screening, need for second screen)
    b. Which type of midwife do you target? (nurse midwives, lay midwives) If more than one group, how do you adjust the education for the different roles?
5- How do you monitor if this education is being delivered to all the midwives in your state?

6- How do you know if the education is working?
7- How often do you review of the midwife education plan? How do you do the review?
    a. How do you decide if you need to make changes or update the plan/materials?

Recommendations around Education

1- If you could change one thing about your current education process, what would you change?

Education for Birthing Centers and Midwives Highlights

Challenges/Barriers/Concerns
Strengths

Section 7: Birth Facilities

This section includes:

- ✓ A list of questions to ask each birth facility

The follow-up experts should be the lead but the rest of the team should also feel free to ask questions.

The goal is to understand how the hospitals handle newborn screening (collection, delivery), education, and communication between the state newborn screening and the hospital.

Checklist of follow-up documents to be reviewed by Site Review Team (SRT):

- ☐ Education materials for parents
- ☐ Education materials used when parent refuses
- ☐ Any education materials provided by the state
- ☐ Any tools used to track timing of blood spot, EHDI, and CCHD

Birthing Facility Summary of Key Points

At the end of touring each hospital you should be able to address if the birthing facility has the following:

- Adequate education for all staff who touch a blood spot kit/card on:
    - Importance of newborn screening
    - Importance of timeliness
    - Blood spot collection
    - Drying time and procedures of blood spots
    - Preparing blood spot cards
    - How to talk to parents about newborn screening

- Bloodspot collection procedures followed according to Clinical Laboratory Standards Institute (CLSI) guidelines.
    - Documentation of those procedures
- Adequate procedures for collecting blood spots and preparing the card for the State Health Lab
- Adequate procedure to gather necessary demographics for blood spot card
- Adequate procedures to track specimens
- Adequate procedures to track which babies have been tested

- Appropriate parent education approach and materials
- Adequate protocol for dealing with and documenting parent refusals

- Adequate procedures to conduct EHDI and CCHD screenings
- Adequate procedures to track EHDI and CCHD results

- Adequate communication system with State Health Lab
    - Number of babies identified
    - Unsats
    - Timeliness of collection and demographics
    - Abnormal results

- Adequate training and education from the state NBS program

- Protocol for dealing with transfer babies (either as senders or receivers)

Birthing Facility #1

Name of Facility ____

Type of Facility number of births _______________________

Birthing Facility #2

Name of Facility ____

Type of Facility number of births _______________________

Sit Down Meeting with Birthing Facility

1- Tell us a little bit about your facility.
   a. How many births do you have each year? _______________
   b. Each month? _________________
2- Tell us about your newborn screening policies and procedures.
   a. Are they written down somewhere? (can we see them?)
   b. How are staff educated on these?
   c. How often are they updated?
   d. How are staff educated on any changes?
3- Can you walk us through preparing the NBS specimen for the state lab?
   a. How do you get the blood spot collection kits?
   b. Who collects the specimen?
   c. When?
   d. How long does it dry?
   e. How are staff trained on this process?
   f. Do you batch specimens?
   g. How is it recorded when the specimen left?
   h. How long is it between you get the specimen and it is shipped to the lab?
4- How does the state report back to you on the number of babies who were identified as having a health issue?
   a. What format do you receive feedback?
   b. How often do you receive feedback?
   c. Who receives feedback?
   d. Who responds to questions about performance from the newborn screening laboratory?
5- In your view, how is the facility's communication with the NBS program?
6- If you could change one thing about the NBS process or your relationship with the state health department, what would you change?

Questions for Well-Baby Nursery Birthing Facility

1- Can you walk us through gathering the blood spots?
   a. Who does it?
   b. When?
   c. How is it recorded?
   d. What steps are in place to assess quality of the specimens?
   e. How staff here are trained on how to get a blood spot sample?
   f. How long does it dry?
   g. How are staff trained on this process?
   h. How is it recorded when the specimen left your unit?

2- How do you keep track of which infants get their newborn screenings tests (blood spot, EHDI, CCHD)?

3- How do you let parents know that their infants will/have undergone newborn screening?

4- What happens when a parent says they do not want a newborn screen?
   a. What is the process to document a refusal?

5- What do you do if a baby leaves before 24 hours?

6- What happens if you have an abnormal result from the blood spot?
   a. How are you notified?
   b. What do you do here after you are notified?

7- Can you walk us through the process of the hearing screening?
   a. Who does it?
   b. When?
   c. How is it recorded?
   d. How are staff trained on how to conduct the EHDI screen?
   e. What steps are in place to assess quality of the test?
   f. What happens if you get an abnormal result?
   g. How do you let the state know the testing results?

8- Can you walk us through the process of Critical Congenital Heart Disease (CCHD) screening?
   a. Who does it?
   b. When?
   c. How is it recorded?
   d. How are staff trained on how to conduct the CCHD screen?
   e. What steps are in place to assess quality of the test?
   f. What happens if you get an abnormal result?
   g. How do you let the state know the testing results?

Questions for the NICU Birthing Facility

1- Can you walk us through preparing the NBS blood spot specimen for the state lab?
   a. Who does it?
   b. When?
   c. How is it recorded?
   d. What steps are in place to assess quality of the specimens?
   e. How staff here are trained on how to get a blood spot sample?
   f. How long does it dry?
   g. How are staff trained on this process?
   h. How is it recorded when the specimen left your unit?
   i. What about second NBS screen (if 2 screen state)

2- How do you keep track of which infants get their newborn screenings tests (blood spot, EHDI, CCHD)?

3- How do you let parents know that their infants will/have undergone newborn screening?

4- What happens when a parent says they do not want a newborn screen?
   a. What is the process to document a refusal?

5- What happens for an infant who is transferred to your facility?
   a. A transfused infant?

6- What happens if you have an abnormal result from the blood spot?
   a. How are you notified?
   b. What do you do here after you are notified?

Questions for Hospital Lab Birthing Facility

If the hospital lab is not involved ask whichever department sends the specimens to the state lab.

1- Can you tell us about the courier you use/how you ship or send specimens?
   a. Who is it?
   b. How often do they pick up samples?
   c. How do you know when samples are received by the lab?

2- What happens if you have an abnormal result from the blood spot?
   a. How are you notified?
   b. What do you do here after you are notified?

3- What happens if you have an unsatisfactory specimen?
   a. are you notified?
   b. What do you do here after you are notified?

4- What feedback do you get from the newborn screening system to assess how well your facility is doing?
   a. How is that shared with the rest of the hospital staff?

Section 8: Medical Providers/Specialists

To fill out this section you should talk to specialists who work with the newborn screening program. This section includes:

- ✓ Questions about:
  - o How they work with the newborn screening program
  - o Communication between the specialists and the NBS program
  - o Perceived Strengths
  - o Perceived Opportunities for Growth

The medical provider/specialist should be the lead but the rest of the team should also feel free to ask questions.

The goal is to understand how the program is working with specialists and their approach is meeting the needs of the specialists.

1- What is your specialty?
    a. How long have you been working with the program?
    b. Are you on the Newborn Screening Advisory board?
2- How do you interact with the state newborn screening program?
    a. How well does this work?
3- How is the communication between you and the program?
    a. How do you track missed cases?
    b. How does the program share successes?
4- In your view, what are the programs' strengths or positives?
5- What are the challenges/opportunities for improvement?
6- What one thing would make your work with the state's newborn screening better?

Section 9: Information System

This section includes:

- ✓ Questions about:
    - o An Overview of State's Information Systems
    - o Perceived Utility of Information System
    - o Use of Data

These questions can be covered at any time during the site visit. Ideally the IT person/dept as well as management in the lab and follow-up should be present to discuss the IT system. Both the laboratory and follow-up sections have information on the state's IT system. If IT was not an issue listed in the PreSite Tool and time is tight, this entire section may not be covered.

The goal is to determine if the IT system allows laboratory and follow-up to easily share information.

The IT/HIT specialist should lead this section. If there is not one on the team, the group should decide who is most comfortable asking these questions.

Information System Summary of Key Points

At the end of this discussion you should be able to address:

- If the lab and follow-up use the same LIMS/IT system.
- If lab feels the HIT set up meets their needs.
- If follow-up feels the HIT set up meets their needs.
- How data is extracted from the system and if that is adequate for the programs.
- If lab and follow-up have remote access in case there is a need to pull data off-site.
- If data can be transferred electronically between the hospitals and the state NBS program.
- If data can be transferred electronically between the healthcare providers and the state NBS program.
    - o What data is being shared
- What HL7 messaging guides are being used (if any)
- Level of technical support

Overview of State's HIT Systems

1- What application is currently in use by:

Component of Program	
Your laboratory	Neometrics/ Natus: Version: ___________<br>StarLims: Version: ___________<br>PerkinElmer: Version: ___________<br>OZ Systems: Version: ___________<br>Internally Developed<br>Other (specify)
Your Short-Term Follow-up	Neometrics/ Natus: Version: ___________<br>StarLims: Version: ___________<br>PerkinElmer: Version: ___________<br>OZ Systems: Version: ___________<br>Internally Developed<br>Other (specify)

2- What other databases do you use, if any? (list name and which group(s) uses that database).
3- Are your NBS laboratory and short-term follow-up program IT applications integrated? (e.g., even if lab and follow-up use a different system, is the lab/follow-up data captured in a single place or would it be entered into the LIMS and a separate spreadsheet)?
4- Describe how do laboratory and follow-up Information Technology systems interact

Perceived Utility of Information System

1- Does your current HIT set up meet the needs of the lab program? (NOTE: This same question was asked with the laboratory staff)
2- Is it meeting the needs of the follow-up program? (NOTE: This same question was asked with the follow-up staff)

Use of Data

1- How do you query your data to answer the questions required to operate your NBS program?
2- Can reports to state, regional and national databases be generated?
  a. Describe the process. Can it be done monthly? Annually? Who manages the queries/reports?
3- What are the challenges with your information system?
4- What type of remote data entry/access does the birth facility have?

5- Can data be transferred electronically to and from birth facilities?
   a. How is it transferred?

6- With whom are you currently transmitting any NBS data electronically?

Partner	Circle correct answers:	Type of NBS Data Shared	Notes
Birthing Hospitals	Fully automatic<br>Partially automatic<br>Manual	o NBS Orders<br>o NBS Results<br>o Other (specify)	
Physicians	Fully automatic<br>Partially automatic<br>Manual	o NBS Orders<br>o NBS Results<br>Other (specify)	

With whom are you currently transmitting any NBS data electronically?

Partner	Circle correct answers:	What Data is Being Shared?
Other State NBS Programs	Fully automatic<br>Partially automatic<br>Manual	
Vital Records	Fully automatic<br>Partially automatic<br>Manual	
Immunization Registry	Fully automatic<br>Partially automatic<br>Manual	
EHDI Database	Fully automatic<br>Partially automatic<br>Manual	
CCHD Database	Fully automatic<br>Partially automatic<br>Manual	
Birth Defects Registry	Fully automatic<br>Partially automatic<br>Manual	
Short-term Follow-up	Fully automatic<br>Partially automatic<br>Manual	
Long-term Follow-up	Fully automatic<br>Partially automatic<br>Manual	
Health Information Exchange	Fully automatic<br>Partially automatic<br>Manual	

Partner	Circle correct answers:	What Data is Being Shared?
NewSTEPs	Fully automatic<br>Partially automatic<br>Manual	
NBSTRN-LPDR	Fully automatic<br>Partially automatic<br>Manual	
NBSTRN-R4S	Fully automatic<br>Partially automatic<br>Manual	
NBSTRN-VRDBS	Fully automatic<br>Partially automatic<br>Manual	
Regional Collaborative Databases	Fully automatic<br>Partially automatic<br>Manual	

7- If using HL7 messaging, what HL7 messaging guide did you use for the implementation?

HL7 messaging guides
PHII NBS HL7 2.3.1 Results implementation guide
PHII NBS HL7 2.5.1 Order implementation guide
PHII NBS HL7 2..5.1 Results implementation guide
HRSA/NLM annotated HL7 v2.5.1 example Results message
Other (specify)

8- Is there HL7 messaging between the NBS data system(s) and healthcare facilities?
   a. Is National Library of Medicine standardized messaging used?
   b. If not, what are the barriers?
9- How are you submitting data to the NewSTEPs Data Repository? [using CSV file transfer?] Is there something that can be done to make this easier?
10- Where in your program do you go for technical expertise when you have a problem related to HIT or data questions?

Recommendations around the Information System(s)

1- What one change could make your job easier?

Information System Highlights

Challenges/Barriers/Concerns
Strengths

Section 10: Next Steps

Each state having an evaluation site visit will receive a comprehensive, written report of site review findings approximately 4 months following the visit.

State program staff is welcome to reach out to NewSTEPs at any time if they have additional questions or concerns.

NewSTEPs Staff Point of Contact:
Ruthanne Salsbury, MPH
Specialist, NewSTEPs
Association of Public Health Laboratories
8515 Georgia Avenue, Suite 700
Silver Spring, MD 20910
T: 240-485-2767
F: 240-285-2700
E: Ruthanne.Salsbury@aphl.org
